# S-100B and neuron-specific enolase as predictors of neurological outcome in patients after cardiac arrest and return of spontaneous circulation: a systematic review

**DOI:** 10.1186/cc7973

**Published:** 2009-07-22

**Authors:** Koichiro Shinozaki, Shigeto Oda, Tomohito Sadahiro, Masataka Nakamura, Yo Hirayama, Ryuzo Abe, Yoshihisa Tateishi, Noriyuki Hattori, Tadanaga Shimada, Hiroyuki Hirasawa

**Affiliations:** 1 Department of Emergency and Critical Care Medicine, Graduate School of Medicine, Chiba University, 1-8-1 Inohana, Chuo-ku, Chiba City, 260-8677, Japan

## Abstract

**Introduction:**

Neurological prognostic factors after cardiopulmonary resuscitation (CPR) in patients with cardiac arrest (CA) as early and accurately as possible are urgently needed to determine therapeutic strategies after successful CPR. In particular, serum levels of protein neuron-specific enolase (NSE) and S-100B are considered promising candidates for neurological predictors, and many investigations on the clinical usefulness of these markers have been published. However, the design adopted varied from study to study, making a systematic literature review extremely difficult. The present review focuses on the following three respects for the study design: definitions of outcome, value of specificity and time points of blood sampling.

**Methods:**

A Medline search of literature published before August 2008 was performed using the following search terms: "NSE vs CA or CPR", "S100 vs CA or CPR". Publications examining the clinical usefulness of NSE or S-100B as a prognostic predictor in two outcome groups were reviewed. All publications met with inclusion criteria were classified into three groups with respect to the definitions of outcome; "dead or alive", "regained consciousness or remained comatose", and "return to independent daily life or not". The significance of differences between two outcome groups, cutoff values and predictive accuracy on each time points of blood sampling were investigated.

**Results:**

A total of 54 papers were retrieved by the initial text search, and 24 were finally selected. In the three classified groups, most of the studies showed the significance of differences and concluded these biomarkers were useful for neurological predictor. However, in view of blood sampling points, the significance was not always detected. Nevertheless, only five studies involved uniform application of a blood sampling schedule with sampling intervals specified based on a set starting point. Specificity was not always set to 100%, therefore it is difficult to indiscriminately assess the cut-off values and its predictive accuracy of these biomarkers in this meta analysis.

**Conclusions:**

In such circumstances, the findings of the present study should aid future investigators in examining the clinical usefulness of these markers and determination of cut-off values.

## Introduction

Identifying neurological prognostic factors after cardiopulmonary resuscitation (CPR) in patients with cardiac arrest (CA) as early and accurately as possible is urgently needed to determine therapeutic strategies after successful CPR and avoid medical futility. Many investigators have previously attempted to establish them [1,2].

Epidemiological data on CA are generally accumulated according to the Utstein Templates [3-5], and retrospective analysis of these data allows, to a certain extent, prognostic prediction of the patients following CA [6,7]. In fact, it is difficult to decide on therapeutic measures for a particular patient based solely on information obtained using the Utstein Templates, due to limitations including: the scope of these templates for registry of cases of CA are limited to individuals with disease categorized as 'cardiac etiology' and 'witnessed arrest; and the specificity of prognostic parameters examined is not satisfactory [1].

Although most of the data specified in the Utstein Templates are collected prior to 'return of spontaneous circulation' (ROSC), data obtained after ROSC also provide valuable information on the prognosis of individual patients. Therefore, the latest version of the Utstein Templates highlighting post-resuscitative care recommends development of a special template for collection and recoding of data in the post-resuscitation phase [8]. Neuroimaging data (e.g., brain computed tomography and magnetic resonance imaging), electrophysiological data (e.g., electroencephalography, auditory brainstem response recording, and somatosensory evoked potential recording), and blood or cerebrospinal fluid (CSF) examination data (i.e., levels of proteins specific to the central nervous system) [1,2] have been tested for clinical usefulness as neurological prognostic predictors by many investigators, although they are all classified as 'supplementary data' in the latest version of the Utstein Templates.

Neuroimaging requires transfer of the patient to a dedicated examination site equipped with special devices, which makes it difficult to apply it to individuals who are receiving high doses of inotropes or circulatory support with an extracorporeal device. Acquisition and analysis of electrophysiological data requires qualified specialists in particular medical fields, making it difficult to perform such examinations in individuals presenting after weekday clinic hours. In contrast, laboratory data are easily obtained with high reproducibility as a part of normal intensive care routine, a feature favorable for clinical application. Considering the extent of insult associated with sample collection, blood may be preferable to CSF.

Serum levels of protein neuron-specific enolase (NSE) and S-100B are considered promising candidates for neurological prognostic predictors in patients with ROSC after CPR, and many investigations on the clinical usefulness of these biochemical markers in predicting neurological outcomes after CPR have been published [1,9,10]. In the present study, we performed an extensive literature review to examine the clinical usefulness of NSE and S-100B as post-resuscitation neurological prognostic predictors.

To improve applicability of study results in clinical practice, we considered the following three points when designing the present study: a consistent definition of poor (good) outcome should be used in assessing data from multiple studies; the cut-off values for biochemical markers should be set so that specificity in prediction of poor outcome is 100%; and the time points of blood sampling should be fixed in assessing the time course of change in blood levels of biochemical markers. Although few reviews of application of biological markers to prediction of neurological outcome in CA after CPR published meet the above requirements [1,10], the present study is the first extensive literature review that does meet them.

## Materials and methods

### Literature search

A Medline search of literature published before August 2008 was performed using the following search terms: 'neuron-specific enolase and cardiac arrest', 'neuron-specific enolase and cardiopulmonary resuscitation', 'NSE and cardiac arrest', and 'NSE and cardiopulmonary resuscitation' with respect to NSE, and 'S100 and cardiac arrest' and 'S100 and cardiopulmonary resuscitation' with respect to S-100B. Cross-references were retrieved from the studies and reviews thus identified. The search included all types of publications (reviews, original studies, case reports, and editorials), but excluded those not in English and animal experimental studies. One author (KS) performed the selection and reviewed all full-text papers.

### Selection of studies

Publications examining the clinical usefulness of NSE or S-100B as a prognostic predictor in two outcome groups, 'favorable outcome' and 'poor outcome', were reviewed, with case reports excluded at this stage. When a study examined a prognostic predictor (or predictors) other than NSE and S-100B as well, only the results for NSE and/or S-100B were reviewed.

### Definition of outcome

Cerebral performance was evaluated according by Cerebral Performance Categories (CPC) 1 to 5 of the Glasgow-Pittsburgh Outcome Categories [11] and the Glasgow Outcome Scale [12,13] (GOS) scores 1 to 5, as recommended by the Utstein Template [4]. The relations between the corresponding grades in the two different grading systems were considered to be as follows. CPC 1 ('good cerebral performance: conscious and alert with normal neurological function or only slight cerebral disability') was equivalent to GOS 5 ('good recovery: able to return to work or school'). CPC 2 ('moderate cerebral disability: conscious and sufficient cerebral function for part-time work in sheltered environment or independent activities of daily life') was equivalent to GOS 4 ('moderate disability: able to live independently but unable to return to work or school'). CPC 3 ('severe cerebral disability: conscious and dependent on others for daily support because of impaired brain function') was equivalent to GOS 3 ('severe disability: able to follow commands but unable to live independently'). CPC 4 and GOS 2 (both defined as 'coma, vegetative state') were mutually equivalent. CPC 5 and GOS 1 (both defined as 'dead or brain dead') were mutually equivalent.

When a study to be reviewed did not involve evaluation of cerebral performance according to either CPC or GOS, one author (KS) assigned the grade that appeared most appropriate as judged from the description in the original article. The actual time point of assessment of cerebral performance as study endpoint, which varied from study to study, was used in the present review as it was in the original text.

In general, outcome after CA is defined dichotomously in the following three respects: dead or alive (survival); regained consciousness or remained comatose (consciousness); and return to independent daily life or not.

To fit this dichotomous system of description, the neurological outcome graded in each study according to CPC, GOS, and Glasgow Coma Scale (GCS) was further grouped for the present review as follows: 'dead': died in hospital; 'alive': survival at the endpoint defined in each study; 'remained comatose': CPC 4 or 5, GOS 1 or 2 (persistent coma, GCS score ≤ 7); 'regained consciousness': CPC 1 to 3, GOS 3 to 5 (obeying simple verbal commands, GCS score ≥ 8); 'no return to independent daily life': CPC 3 to 5, GOS 1 to 3; 'return to independent daily life': CPC 1 or 2, GOS 4 or 5.

### Classification of sampling points

The various sampling time points in individual studies were classified into the following six categories for the sake of simplicity: (1) 'on admission': time points described as 'on admission', 'day 0', or 'within 8 hours after CA'; (2) 'day 1': time points described as 'day 1' or 'at or within 12 hours after CA'; (3) '24 hours': time points described as 'at 24 hours ± 4 hours'; (4) 'day 2': time points described as 'day 2', 'at 36 hours', or 'between 24 and 48 hours'; (5) '48 hours': time points described as 'at 48 hours ± 4 hours'; (6) 'day 3': time points described as 'day 3' or 'at 60 hours'.

### Statistical analysis

Results of statistical comparison between two outcome groups are cited in the present study as reported in the original article, with *P *values presented after classification into the following three categories for simplicity: not significant (≥ 0.05); < 0.05; and < 0.01. The cut-off value for serum level of NSE or S-100B predictive of a poor outcome ('dead', 'remained comatose' or 'no return to independent daily life') was cited as being reported in each original article, together with the corresponding values of sensitivity, specificity, and accuracy calculated using a 2 × 2 contingency table. When the original article reported only raw data (*i.e*., serum level of a particular biochemical marker and outcomes of individual patients), we determined the significance of the difference between two outcome groups with the unpaired *t*-test or the Mann-Whitney *U *test as appropriate, and further calculated the cut-off value for serum level of each biochemical marker predictive of poor outcome with a specificity of 100% by receiver-operating characteristics (ROC) analysis. A two-tailed *P *value of < 0.05 was considered significant. All statistical analyses were carried out using SPSS software (SPSS Japan Inc., Tokyo, Japan).

## Results

A total of 54 papers were retrieved by the initial text search, and 29 of them met the selection criteria. After these 29 papers were reviewed in full text and searched for cross-references, 31 papers were finally selected for the present review. The full-text contents of all 31 papers, which included 26 original articles [14-39] and five review articles [1,2,9,10,40], were reviewed and compared. Twenty-one original articles [14,18,21-39] investigated NSE, while 14 [14-20,22,25,26,28,29,31,39] investigated S-100B. Articles by Mussack and colleagues [19] and Hachimi-Idrissi and colleagues [15] were excluded from further review because they reported serum levels of S-100B in patients with CA after CPR but without comparison between different outcome groups. Therefore, we systematically reviewed a total of 24 original articles.

Generally, systematic review articles seemed not to contain any more data or results than original reports. However, inclusion of all previously published papers is one of the main purposes of this study, and therefore all the review articles were subjected to the cross-referencing and those articles were included in this study.

### 'Dead' vs 'Alive'

Four studies [14,18,20,24] investigated the clinical usefulness of NSE and/or S-100B as a prognostic predictor for two outcome groups, 'dead' and 'alive'. Table 1 summarizes the results of statistical comparison of serum levels of each biochemical marker between the two groups. Table 2 indicates cut-off values for individual biochemical markers predicting death with the corresponding values of sensitivity, specificity, and accuracy.

**Table 1:** Comparison of values for biomarkers between dead and alive

Authors	Year	Ref	n	on admission	Day 1	24 hours	Day 2	48 hours	Day 3
**NSE**									
Grubb and colleagues	2007	[14]	143	-	*P *< 0.01	-	*P *< 0.01	-	-
Auer and colleagues	2006	[24]	17	NS	NS	-	*P *< 0.05	-	-
**S-100B**									
Grubb and colleagues	2007	[14]	143	-	*P *< 0.01	-	*P *< 0.01	-	-
Bottiger and colleagues	2001	[18]	66	NM	-	NM	-	NM	-
Rosen and colleagues	1998	[20]	41	-	*P *< 0.01	-	*P *< 0.01	-	*P *< 0.01

NM = not mentioned for statistical comparison; NS = not significance; NSE = neuron specific enolase; Ref = references.

**Table 2:** Values of cutoff points and predictive accuracy for dead

	On admission	Day 1	24 hours	Day 2	48 hours	Day 3
Authors	cut/spe/sen/acc	cut/spe/sen/acc	cut/spe/sen/acc	cut/spe/sen/acc	cut/spe/sen/acc	cut/spe/sen/acc
NSE						
Grubb and colleagues	-	NM	-	(24)/86/60/**72**	-	-
Grubb and colleagues	-	NM	-	(71)/100/14/**54**	-	-
Auer and colleagues	NM	NM	-	(30)/100/79/**88**	-	-
**S-100B**						
Grubb and colleagues	-	NM	-	(0.3)/76/73/**75**	-	-
Grubb and colleagues	-	NM	-	(1.2)/100/45/**74**	-	-
Bottiger and colleagues	(0.2)/**45/100/82**	-	(0.2)/**80/100/91**	-	(0.2)/**70/100/84**	-
Rosen and colleagues	-	(0.2)/**81/77/79**	-	(0.2)/**100/79/92**	-	NM

Values in bold are the results of our calculation. cut = values of cutoff points (ng/mL); spe = specificity (%); sen = sensitivity (%); acc = accuracy (%).

NM = not mentioned for cutoff values and predictive accuracy; NSE = neuron specific enolase.

The clinically useful outcome that can be predicted using NSE and/or S-100B, which are biomarkers specific to the central nervous system, is neurological outcome rather than survival outcome. Consequently, association of these biomarkers with survival outcome was investigated in a limited number of studies. Grubb and colleagues [14] demonstrated in a study involving a relatively large number of subjects (n = 143) that S-100B assayed on day 2 was slightly superior to NSE assayed concomitantly with respect to predictive accuracy for mortality.

### 'Regained consciousness' vs. 'Remained comatose'

Sixteen studies [16,21-23,25,27,28,31-39] investigated the clinical usefulness of NSE and/or S-100B as a prognostic predictor in two outcome groups, 'regained consciousness' and 'remained comatose'. Table 3 summarizes the results of statistical comparison of serum levels of each biochemical marker between the two groups. Table 4 indicates cut-off values for individual biochemical markers predicting persistent coma with the corresponding values of sensitivity, specificity, and accuracy.

**Table 3:** Comparison of values for biomarkers between remained coma and regained consciousness

Authors	Year	Ref	n	on admission	Day 1	24 hours	Day 2	48 hours	Day 3
**NSE**									
Reisinger and colleagues	2007	[21]	227	NM	NM	-	NM	-	NM
Prohl and colleagues	2007	[39]	80	-	*P *< 0.01	-	*P *< 0.01	-	*P *< 0.01
Zandbergen and colleagues	2006	[22]	407	-	-	NM	-	NM	-
Rech and colleagues	2006	[23]	45	-	-	*P *< 0.01	-	-	-
Pfeifer and colleagues	2005	[25]	97	-	*P *< 0.05-	-	*P *< 0.05	-	*P *< 0.05
Meynaar and colleagues	2003	[27]	110	NS	-	*P *< 0.01	-	*P *< 0.01	-
Zingler and colleagues	2003	[28]	27	NS	*P *< 0.05	-	*P *< 0.05-	-	-
Martens and colleagues	1998	[31]	64	-	-	*P *< 0.01	-	-	-
Fogel and colleagues	1997	[32]	50	*P *< 0.01	*P *< 0.01	-	*P *< 0.01	-	*P *< 0.01
Martens	1996	[34]	52	-	-	*P *< 0.01	-	-	-
Bassetti and colleagues	1996	[33]	60	-	NM	NM	-	-	-
Stelzl and colleagues	1995	[35]	13	**NS**	***P < 0.05***	***P < 0.05***	***P < 0.05***	***P < 0.05***	***P < 0.05***
Karkela and colleagues	1993	[36]	20	NS	-	NS	-	-	-
Dauberschmidt and colleagues	1991	[37]	18	**NS**	**NS**	**-**	**NS**	**-**	**NS**
Roine and colleagues	1989	[38]	75	-	-	*P *< 0.01	-	-	-
**S-100B**									
Prohl and colleagues	2007	[39]	80	-	*P *< 0.01	-	*P *< 0.01	-	*P *< 0.01
Zandbergen and colleagues	2006	[22]	407	-	-	NM	-	NM	-
Pfeifer and colleagues	2005	[25]	97	-	NS	-	*P *< 0.05	-	*P *< 0.05
Zingler and colleagues	2003	[28]	27	*P *< 0.05	*P *< 0.01	-	*P *< 0.05	-	-
Hachimi-Idrissi and colleagues	2002	[16]	58	*P *< 0.01	-	*P *< 0.01	-	-	-
Martens and colleagues	1998	[31]	64	-	-	*P *< 0.01	-	-	-

Values in bold are the results of our calculation. NM = not mentioned for statistical comparison; NS = not significance; NSE = neuron specific enolase; Ref = references.

**Table 4:** Values of cutoff points and predictive accuracy for remained coma

	on admission	Day 1	24 hours	Day 2	48 hours	Day 3
Authors	cut/spe/sen/acc	cut/spe/sen/acc	cut/spe/sen/acc	cut/spe/sen/acc	cut/spe/sen/acc	cut/spe/sen/acc
**NSE**						
Reisinger and colleagues	NM	NM	-	NM	-	NM
Prohl and colleagues	-	(29)/100/33/**78**	-	(32)/100/33/**78**	-	(28)/100/67/**89**
Zandbergen and colleagues	-	-	NM	-	NM	-
Rech and colleagues	-	-	(60)/100/35/**49**	-	-	-
Pfeifer and colleagues	-	NM	-	NM	-	(65)/96/40/**56**
Meynaar and colleagues	NM	-	NM	-	NM	-
Zingler and colleagues	(48)/100/53/**70**	(43)/100/91/**94**	-	(91)/100/75/**84**	-	-
Martens [31]	-	-	(20)/89/51/**68**	-	-	-
Fogel and colleagues	(33)/100/25/**56**	(33)/100/60/**77**	-	(33)/100/63/**78**	-	(33)/100/65/**80**
Martens [34]	-	-	(18)/**67/74/71**	-	-	-
Bassetti and colleagues	-	NM	NM	-	-	-
Stelzl and colleagues	**(18)/100/40/67**	**(17)/100/83/92**	**(21)/100/100/100**	**(29)/100/100/100**	**(36)/100/100/100**	**(24)/100/100/100**
Karkela and colleagues	NM	-	NM	-	-	-
Dauberschmidt and colleagues	**(11)/100/43/56**	**(5)/100/50/60**	**-**	**(5)/100/67/78**	**-**	**(5)/100/71/80**
Roine and colleagues	-	-	(17)/98/40/**80**	-	-	-
**S-100B**						
Zandbergen and colleagues	-	-	NM	-	NM	-
Prohl and colleagues	-	(2.1)/100/17/**72**	-	(1.8)/100/17/**72**	-	(1.2)/100/33/**78**
Pfeifer and colleagues	-	NM	-	NM	-	(1.5)/96/34/**51**
Zingler and colleagues	(5.2)/100/35/**59**	(0.8)/100/64/**77**	-	(0.5)/100/75/**84**	-	-
Hachimi-Idrissi and colleagues	(0.7)/85/67/78	-	(0.7)/88/100/93	-	-	-
Martens [31]	-	-	(0.7)/96/55/**73**	-	-	-

Values in bold are the results of our calculation. cut = values of cutoff points (ng/mL); spe = specificity (%); sen = sensitivity (%); acc = accuracy (%).

NM = not mentioned for cutoff values and predictive accuracy; NSE = neuron specific enolase.

In addition to those reporting cut-off values according to time course interval listed in Table 4, the following four studies investigated the clinical usefulness in predicting neurological outcome after CPR not in accordance with time course interval. Reisinger and colleagues [21] assayed serum NSE concentrations at five different time points after CPR (from ICU admission to day 4), although without statistical comparison between different outcome groups, to determine the cut-off value for peak NSE concentration within these five points predictive of 'persistent coma (CPC 4)' with a specificity of 100%. They reported that a peak NSE concentration more than 80 ng/mL (noted by day 4) was invariably associated with 'persistent coma', that is, no patients meeting this criterion regained consciousness. They further concluded, based on the results of ROC analysis, that a cut-off value of 80 ng/mL for peak NSE concentration predicted 'persistent coma' at a specificity of 100% with a sensitivity of 63% and a predictive accuracy of 88%. Zandbergen and colleagues [22] assayed serum NSE and S-100B concentrations at three different time points (24, 48, and 72 hours after CPR) and reported a cut-off value for peak NSE concentration within these three points, 33 ng/mL, corresponding to a positive predictive value of 100% and a cut-off value for peak S-100B concentration of 0.7 ng/mL corresponding to a positive predictive value of 98%. Meynaar and colleagues [27] reported that no patient with a serum NSE level of more than 25 ng/mL at 24 or 48 hours after CPR regained consciousness (specificity 100%, sensitivity 59%). Bassetti and colleagues [33], who determined serum NSE concentrations at two different time points (12 and 24 hours after CPR), referred to a positive predictive value for a serum NSE concentration exceeding the normal level at each time point without calculation of a cut-off value.

The number of studies comparing the two neurological outcome groups, 'remained comatose' vs. 'regained consciousness', was 16, and greater than the number of studies comparing any other pair of outcome groups. Of these 16 studies, seven reported serum NSE levels on admission, while two reported serum S-100B levels on admission. One study (1/7, 14.3%) detected a significant difference in NSE on admission between the two outcome groups, while two studies (2/2, 100%) identified a significant difference in S-100B on admission. On the other hand, five studies (5/7, 71.4%) failed to detect a significant difference between groups in NSE on admission, while no study failed to detect such a difference in S-100B on admission (Table 3). These findings suggest that S-100B assayed on admission may be more useful than NSE assayed concomitantly as an early biochemical predictor of success or failure in regaining consciousness. The reported values of predictive accuracy corresponding to a cut-off value on admission for S-100B prediction of persistent coma with 100% specificity in the study by Zingler and colleagues [28] is a little higher (59%) than that for NSE (56%) in the study by Fogel and colleagues [32] those detected a significant difference between the two outcome groups.

At no sampling time point other than 'on admission' any particular tendency was noted with respect to the clinical usefulness of NSE and S-100B as neurological prognostic predictors.

### 'Return to independent daily life' vs. 'no return to independent daily life'

Five studies [17,18,26,29,30] investigated the clinical usefulness of NSE and/or S-100B as a prognostic predictor in two outcome groups, 'return to independent daily life' and 'no return to independent daily life'. Table 5 summarizes the results of statistical comparison of serum levels of each biochemical marker between the two groups. Table 6 indicates cut-off values for individual biochemical markers predicting no-return to independent daily life with the corresponding values of sensitivity, specificity, and accuracy. Tiainen and colleagues [26] divided their study subjects into two treatment groups, 'hypothermia' and 'normothermia' (not undergoing hypothermia), and then investigated the prognostic values of both biochemical markers in each group.

**Table 5:** Comparison of values for biomarkers between no-return and return to independent daily life

Authors	Year	Ref	n	on admission	Day 1	24 hours	Day 2	48 hours	Day 3
**NSE**									
Tiainen and colleagues (hypothermia)	2003	[26]	36	-	-	NM	NM	NM	-
Tiainen and colleagues (normothermia)	2003	[26]	34	-	-	NM	NM	NM	-
Rosen and colleagues	2001	[29]	66	-	*P *< 0.05	-	*P *< 0.01	-	*P *< 0.01
Bottiger and colleagues	2001	[18]	66	NS	-	*P *< 0.05	-	*P *< 0.05	-
Schoerkhuber and colleagues	1999	[30]	56	NS	*P *< 0.01	*P *< 0.05	-	*P *< 0.01	-
**S-100B**									
Tiainen and colleagues (hypothermia)	2003	[26]	36	-	-	NM	NM	NM	-
Tiainen and colleagues (normothermia)	2003	[26]	34	-	-	NM	NM	NM	-
Mussack and colleagues	2002	[17]	20	NM	NM	-	-	-	-
Rosen and colleagues	2001	[29]	66	-	*P *< 0.01	-	*P *< 0.01	-	*P *< 0.01
Bottiger and colleagues	2001	[18]	66	*P *< 0.05	-	*P *< 0.05	-	*P *< 0.05	-

NM = not mentioned for statistical comparison; NS = not significance; NSE = neuron specific enolase, Ref = references.

**Table 6:** Values of cutoff points and predictive accuracy for no-return to independent daily life

	on admission	Day 1	24 hours	Day 2	48 hours	Day 3
Authors	cut/spe/sen/acc	cut/spe/sen/acc	cut/spe/sen/acc	cut/spe/sen/acc	cut/spe/sen/acc	cut/spe/sen/acc
**NSE**						
Tiainen and colleagues (hypothermia)	-	-	(31)/96/22/**76**	(26)/96/30/**79**	(25)/96/25/**77**	-
Tiainen and colleagues (normothermia)	-	-	(13)/100/59/**80**	(13)/100/63/**82**	(9)/100/76/**88**	-
Rosen and colleagues	-	(25)/**100/14/44**	-	(23)/**100/34/59**	-	(13)/**100/44/69**
Bottiger and colleagues	NM	-	NM	-	NM	-
Schoerkhuber and colleagues	NM	(39)/100/17/**59**	(40)/100/8/**55**	-	(25)/100/48/**75**	-
Schoerkhuber and colleagues	NM	(15)/72/70/**71**	(18)/80/56/**68**	-	(16)/79/72/**76**	-
**S-100B**						
Tiainen M and colleagues (hypothermia)	-	-	(0.21)/100/30/**81**	(0.2)/96/20/**76**	(0.23)/96/22/**76**	-
Tiainen M and colleagues (normothermia)	-	-	(0.19)/100/59/**80**	(0.5)/100/18/**59**	(0.12)/100/88/**94**	-
Mussack and colleagues	NM	(0.76)/100/54/**62**	-	-	-	-
Rosen and colleagues	-	(0.4)/**85/62/70**	-	(0.2)/**100/67/80**	-	(0.19)/**100/40/66**
Bottiger and colleagues	NM	-	NM	-	NM	-

Values in bold are the results of our calculation. cut = values of cutoff points (ng/mL); spe = specificity (%); sen = sensitivity (%); acc = accuracy (%).

NM = not mentioned for cutoff values and predictive accuracy; NSE = neuron specific enolase.

Of the five studies mentioned above, two reported serum NSE levels on admission, while two reported serum S-100B levels on admission. No study detected a significant difference in NSE on admission between the two outcome groups, while one study (1/2, 50%) identified a significant difference between them in S-100B on admission. On the other hand, two studies (2/2, 100%) reported a non-significant difference between groups in NSE on admission, while no study reported such a difference in S-100B on admission (Table 5). These findings suggest that S-100B assayed on admission may be more useful than NSE assayed concomitantly as an early biochemical predictor of return or no-return to independent daily life.

At no sampling time point other than 'on admission' was any particular tendency noted with respect to the clinical usefulness of NSE and S-100B as neurological prognostic predictors.

### Study design

Five of the studies reviewed in this article involved uniform application of a blood sampling schedule to all subjects with sampling intervals specified based on a set starting point (e.g., onset of CA, CPR initiation, or ROSC): Auer and colleagues [24] (n = 17), Tiainen and colleagues [26] (n = 36, 34), Bottiger and colleagues [18] (n = 66), Bassetti and colleagues [33] (n = 60) and Karkela and colleagues [36] (n = 20). However, the sample sizes of these studies were not large. In contrast, four studies including larger numbers of subjects (Grubb and colleagues [14] (n = 143), Reisinger and colleagues [21] (n = 227), Zandbergen and colleagues [22] (n = 407), and Meynaar and colleagues [27] (n = 110)) involved blood sampling as a part of normal intensive care routine with no attention focused on the intervals of sampling points from onset of CA. Many previous studies demonstrated time-dependent changes in blood levels of these biochemical markers after CA [18,21,24,25,28,30,32]. In particular, Bottiger et al. [18] investigated the changes in serum S-100B level within 24 hours after CA in detail, and demonstrated that the serum S-100B level varied every hour.

Assessment of the clinical usefulness of S-100B and NSE in predicting post-resuscitative neurological outcome thus requires a study design with particular attention focused on the intervals of sampling points from the onset of CA, although no multicenter prospective study using such a study design has been published to date.

## Discussion

Biochemical markers in blood samples can be expected to serve as prognostic predictors of CA patient outcome after CPR and be more easily applicable to clinical practice than neuroimaging or electrophysiological findings. In the present study, we performed a systematic literature review to examine the clinical usefulness of NSE and S-100B (proteins specific to the central nervous system and potential biochemical markers of brain damage) as post-resuscitative predictors of neurological prognosis.

Grubb and colleagues [14] performed the multiple logistic regression analysis on mortality in these biomarkers and clinical scores (i.e., arrest rhythm, bystander CPR and GCS score). They showed that NSE was an independently significant predictor among them. Pfeifer and colleagues [25] compared the neurological predictive value between these biomarkers and the clinical predictors, such as time of anoxia, GCS score, presence of bystander CPR, and so on. Prohl and colleagues [39] also compared this value using clinical examination score reflected some brain stem reflexes. Both studies showed the odds ratio of these clinical predictors was lower than those of NSE and S-100B. Those results indicate that predictive value of biomarkers was superior to that of clinical predictors. Clinical predictors were often affected by the clinical situations (e.g., using of sedative agents, relying on information of emergency medical service personnel who collected information at a chaotic emergency scene). Therefore, the specificity of biomarkers is higher than that of clinical predictors, even though the clinical predictors can be more easily assessed.

NSE is the neuronal form of the cytoplasmic glycolytic enzyme enolase. It is a dimeric enzyme composed of two γ subunits (γγ isomer) with a total molecular weight of 78 kDa and a biological half-life of 24 hours. It is mainly located in neurons and neuroendocrine cells [41,42]. The S-100 protein is a calcium-binding protein with a total molecular weight of 21 kDa and a biological half-life of two hours. S-100B, a homodimer composed of two β subunits (ββ form), is secreted from glial cells and Schwann cells [43]. Recent studies have suggested that elevated levels of S-100B might cause neuronal apoptosis, suggesting that S-100B may play a role as a cytokine in brain inflammatory responses [44,45]. Accordingly, in patients with high serum levels of S-100B after CPR, S-100B when present at high levels in the brain is suspected to induce brain cell apoptosis leading to aggravation of post-CA brain injury. S-100B is also considered a putative 'Alarmin' released during an early stage of the inflammatory response [46].

The findings of the present study suggest that, when assayed 'on admission' (i.e., within eight hours after CA), serum levels of S-100B might be more clinically useful than those of NSE in predicting neurological outcomes such as regaining consciousness and returning to independent daily life. Assay of serum S-100B level focuses on the process of aggravation of brain injury, while brain imaging, physical examination, and electrophysiology all focus on the consequences of brain injury. NSE is a protein located in nerve cells and detectable in body fluids as a marker enzyme indicative of nerve cell injury [47]. Monitoring for increases in the serum NSE level thus focuses on cell death as a result of post-CA brain injury. Consequently, S-100B serves as a prognostic predictor within 24 hours after CA, and thus at an earlier stage than other factors (including NSE), which focus on the consequences of brain injury and are therefore meaningful as prognostic predictors one to three days after CA (i.e., only after manifestation of brain injury is completed) [1,22].

Many preceding studies recognized an increase in serum NES level over time in patients with poor outcome after CPR, and also demonstrated a decrease in serum S-100B level over time in those with favorable outcome [14,21,25,26,28-30]. In those studies, these changes were ascribed to the difference in biological half-life between these two proteins. However, it should be noted that these changes can also be explained by considering S-100B a cause of post-CA brain injury and NSE, an enzyme released from nerve cells, as a result of brain injury: NSE as a marker of brain cell injury increases over time and accumulates as a result of aggravation of brain injury, while persistence of S-100B as a cause of brain injury at high levels leads to aggravation of disease, a conclusion consistent with the previously reported findings.

Therefore, we emphasize that the time-course change in early phase of serum S-100B and NSE levels would offer a more reliable indication of what is happening in the brain of the CA patient, which might be useful for optimization of therapeutic intervention in future cases of CA. However, there are few investigations on the time-course of these biomarkers. Usui and colleagues [48] reported the detailed time-course of these biomarkers in serum and cerebrospinal fluid every one to two hours within 18 hours after CA in mongrel dog models. Bottiger and colleagues [18] reported the time-course of human serum S-100B every 15 minutes within 1 hour and at 2, 8, and 24 hours after CA. However, this study by Bottiger and colleagues is limited by the number of subjects. Therefore, there is no investigation on the time-course of these biomarkers involving a large number of human subjects. The future investigators should put more weight on the time-course change of the biomarkers, especially the changes in early phase (i.e., within 24 hours following CA).

The present study identified 24 original articles involving investigation of the clinical usefulness of NSE and S-100B as prognostic predictors of CA patients after CPR. The design adopted varied from study to study, making a systematic literature review extremely difficult [10,40]. The major problems encountered during the review process involved variation or heterogeneity among studies in the following three respects: definitions of outcome to be assessed (e.g., some studies adopted grouping criteria for outcome different from others); the value of specificity corresponding to each cut-off value reported for a particular biochemical marker predictive of a poor prognosis (i.e., not always fixed at 100%); and specification of blood sampling time points (i.e., not always uniform application of a blood sampling schedule to all subjects with sampling intervals specified).

Consistency in the three respects noted above had not been considered in the five review articles previously published on the same subject [1,2,9,10,40], and the present review is the first attempt to consider it.

A recently published article discusses pitfalls in critical care meta-analysis [49], highlighting publication bias and trial-level heterogeneity as pitfalls to be carefully avoided in a systematic review of observational studies such as the present review. To avoid publication bias, we performed an extensive literature search to identify all papers published previously on the clinical usefulness of NSE and S-100B in prediction of prognosis after resuscitation from CA and closely reviewed the full-text contents of all papers thus identified. Trial-level heterogeneities encountered in papers reviewed in the present study include grouping criteria for outcome (definitions of outcome), specification of time points for blood sampling, and assay procedures for individual biochemical markers of interest. Consistency in definitions of outcome and specification of time points for blood sampling appears to be particularly important in the present literature review investigating the clinical usefulness of serum levels of NSE and S-100B as prognostic predictors.

Rapid interdisciplinary treatment and monitoring are required in the early post-resuscitative period, sometimes including percutaneous coronary intervention as well. Consequently, a study design involving uniform blood sampling within a few hours after resuscitation would be difficult to adopt in a multicenter study. Rosen and colleagues [29] studied 66 out-of-hospital CA and collected blood samples at various time points according to their ward routines. Their mean first sample times (± standard error) were 10.5 ± 0.9 hours after CA. In consideration of these results, we think that blood sampling at least once between 4 and 12 hours after resuscitation would be practicable and adoptable. Therefore, a multicenter prospective study involving blood sampling between 4 and 12 hours after resuscitation at a time point specified by interval from onset of CA would be most helpful in investigating the clinical usefulness of S-100B and NSE as early predictors of neurological outcome of CA patients after CPR.

The present review, which included all previous papers identified in a literature search, included a paper published in 1989 [38] as the earliest published report. In the past 20 years, however, techniques of assay for both NSE and S-100B have been greatly improved, with concomitant increase in sensitivity of detection [50,51]. It is therefore difficult, and even inappropriate, to assess the cut-off values reported for serum levels of these biochemical markers during this period using a uniform scale or standard.

Finally, we emphasize that extracellular S100B at μM concentration is harmful to astrocyte and neurons but at nM concentrations is beneficial to those [45,52]. Thus, at least at the very beginning of brain injury the secretion and release of S100B (and hence elevation of serum S100B levels, if any) might not necessarily be indicative of aggravation of brain injury; it may be indicative of activation of astrocytes and attempt to provide neurons with a trophic factor. Thus, it may be important that the levels of serum S100B and NSE be measured at the very onset of CA and at intervals during the next few hours. An analysis of the time-course of serum S100B and NSE levels would offer a more reliable indication of what is going on in the brain of the patient, which might be useful for optimization of therapeutic intervention in future cases.

## Conclusions

The present study shows that the measurements of serum levels of S100B within 24 hours after CA might be clinically more relevant than those of NSE in predicting neurological outcomes.

As noted above, no systematic literature review has been performed including all previously published papers on the clinical usefulness of NSE and S-100B as neurological prognostic predictors. In such circumstances, the findings of the present study should aid future investigators in examining the clinical usefulness of these biochemical markers and determination of cut-off values predictive of poor neurological outcome.

## Key messages

• A consistent definition of poor (good) outcome should be used in assessing data from multiple studies.

• The cut-off values for neurological predictors after CPR should be set so that specificity in prediction of poor outcome is 100%.

• The time points of blood sampling should be fixed in assessing the time course of change in blood levels of NSE and S-100B.

• S-100B assayed on admission may be more useful than NSE assayed concomitantly as an early biochemical predictor of remaining comatose and no-return to independent daily life.

• A multicenter prospective study involving blood sampling after resuscitation at a time point specified by interval from onset of CA would be most helpful in investigating the clinical usefulness of S-100B and NSE as early predictors of neurological outcome.

## Abbreviations

CA: cardiac arrest; CPC: cerebral performance categories; CPR: cardiopulmonary resuscitation; CSF: cerebrospinal fluid; GCS: Glasgow coma scale; GOS: Glasgow outcome scale; NSE: neuron-specific enolase; ROC: receiver-operating characteristics; ROSC: return of spontaneous circulation.

## Competing interests

The authors declare that they have no competing interests.

## Authors' contributions

KS conceived and designed the study. OS and HH made critical revision of the manuscript for important intellectual content. TS, MN, YH, RA, YT, NH, and TS drafted the manuscript. All authors read and approved the final manuscript.

## Authors' information

KS: Clinical Fellow, Department of Emergency and Critical Care Medicine, Chiba University Hospital, Board Certified Member of Japanese Society of Intensive Care Medicine.

OS: Professor and Chairman, Department of Emergency and Critical Care Medicine, Chiba University Graduate School of Medicine, Board Certified Member of Japanese Society of Intensive Care Medicine.

HH: Professor Emeritus and Former Chairman, Department of Emergency and Critical Care Medicine, Chiba University Graduate School of Medicine, Immediate Past President of Japanese Society of Intensive Care Medicine
